# Tumor control by intravenous administration of STING ligand requires combination with precisely timed radiation therapy

**DOI:** 10.1186/2051-1426-3-S2-P349

**Published:** 2015-11-04

**Authors:** Jason Baird, David Friedman, Benjamin Cottam, Marka Crittenden, Michael Gough

**Affiliations:** 1 Earle A. Chiles Research Institute, Providence Cancer Center, Portland, OR, USA; 2 Earle A. Chiles Research Institute, Providence Cancer Center; The Oregon Clinic, Portland, OR, USA

## 

We have demonstrated that radiation alone is weakly effective at generating anti-tumor adaptive immune responses despite release of tumor-associated antigen. We hypothesized that providing potent adjuvant would improve this immune response increasing immune control of local and distant disease. Recently, we have shown that activation of STimulator of INterferon Genes (STING) innate signaling has the ability to propagate an adaptive immune response that is initiated in the tumor microenvironment (TME). Our experiments thus far have applied local administration of STING ligands to the tumor; however, this may not always be practical in patients. In the current work, we evaluated systemic administration of STING-ligand and found that unlike direct intratumoral administration, systemic administration was unable to control a selection of murine tumor models. However, combination with tumor targeted radiation therapy in these models resulted in tumor control by systemic STING-ligands. This effect was dependent on the precise timing of STING-ligand and tumor-targeted radiation. We propose that radiation therapy targets immune cells and inflammatory mechanisms activated by systemic STING-ligand to the tumor environment, resulting in therapeutic synergy.

